# Optimizing Preclinical Models for Oral Cancer: The Influence of 4NQO Administration Routes on Tumor Development

**DOI:** 10.3390/cancers17132108

**Published:** 2025-06-23

**Authors:** Jolien Van den Bosch, Nuran Caz, Sandrina Martens, Céline Erens, Leen Rasking, Pascal Gervois, Kim Nijsten, Uwe Himmelreich, Sofie Van Cauter, Lisa M. Hillen, Herbert Plasschaert, Ivo Lambrichts, Esther Wolfs

**Affiliations:** 1 Laboratory for Functional Imaging and Research on Stem Cells (FIERCE Lab), Biomedical Research Institute (BIOMED), Hasselt University, 3950 Diepenbeek, Belgium; jolien.vandenbosch@uhasselt.be (J.V.d.B.); nuran.caz@uhasselt.be (N.C.); sandrina.martens@uhasselt.be (S.M.); cerens@innoserlaboratories.com (C.E.); pascal.gervois@uhasselt.be (P.G.); kim.nijsten@uhasselt.be (K.N.); 2 Centre for Environmental Sciences, 3950 Diepenbeek, Belgium; 3 Limburg Clinical Research Center (LCRC), 3950 Diepenbeek, Belgium; 4 Biomedical MRI Unit, Department of Imaging & Pathology, KU Leuven, 3000 Leuven, Belgium; uwe.himmelreich@kuleuven.be; 5 Biomedical Research Institute (BIOMED), Faculty of Medicine and Life Sciences, Hasselt University, 3950 Diepenbeek, Belgium; sofie.vancauter@zol.be; 6 Department of medical imaging, Ziekenhuis-Oost Limburg (ZOL), 3600 Genk, Belgium; 7 Department of Pathology, GROW School for Oncology and Reproduction, Maastricht University Medical Center+, 6211 Maastricht, The Netherlands; lisa.hillen@mumc.nl; 8 Department of Pathology, Ziekenhuis-Oost Limburg (ZOL), 3600 Genk, Belgium; herbert.plasschaert@zol.be; 9 Lab for Histology and Regeneration (HISTOREGEN Lab), Biomedical Research Institute (BIOMED), Hasselt University, 3950 Diepenbeek, Belgium; ivo.lambrichts@uhasselt.be

**Keywords:** oral squamous cell carcinoma, 4-Nitroquinoline 1-oxide, rat model, histopathology, ex vivo MRI

## Abstract

Background/Objectives: Oral squamous cell carcinoma (OSCC) is the most common oral cancer, progressing from hyperplasia to dysplasia, carcinoma in situ (CIS), and finally invasive squamous cell carcinoma (ISCC). Developing an animal model that mimics both early and advanced OSCC stages has been challenging. The 4-Nitroquinoline 1-oxide (4NQO) model is considered one of the most suitable, as it represents all stages of OSCC. Nevertheless, thoroughly understanding the properties of the 4NQO model is essential for preclinical testing of novel therapeutics. Methods: We aimed to characterize the 4NQO rat model using two application methods—drinking water and topical application—over eight months. Monthly sacrifices allowed histopathological analysis and ex vivo magnetic resonance imaging (MRI) to track tumor progression. Results: CIS was observed at three months in the drinking water group, evolving into ISCC by six months, while topical application induced CIS at eight months without ISCC formation. The tongue was divided into three regions and histological properties, lesion size, and invasion depth were analyzed. In the drinking water group, particularly in the body of the tongue, we saw earlier CIS development, larger lesions, and deeper invasion. Additionally, assessment of proliferative properties showed an increased cell division in dysplastic lesions that reduced upon invasion. MRI was able to show macroscopic tumoral lesions, in concordance with histology. Conclusions: Overall, the drinking water method closely mimics human OSCC, validating the 4NQO model for translational OSCC research.

## Simple Summary

Understanding how oral cancer develops and progresses is essential to improve treatment strategies. This study focuses on a commonly used animal model for oral cancer that closely mimics the stages of human disease, from early tissue changes to invasive tumors. By tracking tumor growth over time, researchers can identify key moments when treatment may be most effective. However, without careful observation, important aspects of tumor evolution may be overlooked. By combining MRI and microscopic analysis at different time points, tumor development can be tracked. This approach enables assessment of disease progression, helping refine the use of this model in preclinical research.

## 1. Introduction

Oral cancers account for approximately 400,000 new cases and 200,000 deaths each year, making them a significant global health burden [1]. A significant subset of these cases is oral squamous cell carcinoma (OSCC), which originates from the stratified squamous epithelium of the oral cavity and the oropharynx, representing around 90% of all oral neoplasms [2]. The pathogenesis of OSCC typically begins with hyperplasia, further developing into dysplasia, a premalignant condition characterized by the loss of normal epithelial maturation and stratification and the presence of cellular atypia [3]. As the disease advances, dysplasia may evolve into carcinoma in situ (CIS) and further progress into invasive squamous cell carcinoma (ISCC) [1,4].

Several lifestyle factors contribute to the development of OSCC, with excessive tobacco use and alcohol consumption being the primary risk factors. While human papillomavirus infection is a well-established cause of oropharyngeal cancer, its role in OSCC is limited to a small subset of cases [5,6]. Managing OSCC remains challenging, as early-stage disease is often asymptomatic and thus prone to delayed detection. Although public awareness and diagnostic capabilities have improved over the past two decades [7,8,9], a substantial proportion of cases are still diagnosed at an advanced stage [10,11,12]. Despite the range of available therapies including surgery, radiotherapy, chemotherapy, and immunotherapy, the five-year survival rate remains as low as approximately 60% [5,13,14,15]. This underscores the urgent need for new therapies and early diagnostic techniques, which can only be investigated in clinically relevant animal models that replicate the complexity of OSCC in humans. Such models are crucial to advance our understanding of OSCC pathogenesis and to test novel therapeutic strategies aimed at improving patient outcomes [16].

A range of animal models are employed to investigate the pathogenesis of OSCC and potential therapies. These include xenograft, genetic, and chemically induced models, each with distinct advantages and limitations. Xenograft models are known for their high reproducibility and ease of induction but fail to fully replicate human tumor development and the complexity of the tumor microenvironment [17,18]. Recently, genetic models have garnered considerable interest, such as the overexpression of cyclin D1 in a p53-deficient mouse, which leads to oral-esophageal squamous epithelial cancers [18,19,20]. Moreover, inducible mouse models using Cre-Lox systems enable precise control over oncogene activation or tumor suppressor inactivation [18,21]. Despite their promise, these models often fail to replicate key aspects of OSCC, including tissue-specific tumor microenvironment interactions [18,19,22].

One of the first models used in OSCC research is the polycyclic hydrocarbon 9,10 dimethyl-1,2,benzanthracene (DMBA) model in hamster cheek pouches, where the carcinogen is applied on the cheeks over a prolonged period. While this model successfully induces later stages of OSCC, it presents significant drawbacks. The repeated application of DMBA causes inflammation and necrosis, making it unsuitable to study early-onset lesions [23,24]. Additionally, the labor-intensive nature of the model and the differences between hamster and human oral epithelium limit its clinical relevance [16,25].

Given that tobacco use is a primary risk factor for OSCC, a carcinogen that mimics the effects of tobacco compounds provides a more relevant model for oral cancer research. Hence, 4-Nitroquinoline 1-oxide (4NQO) has been widely adopted since the 1960s [26,27]. 4NQO induces oxidative stress, primarily through the generation of reactive oxygen species [28,29], which leads to DNA damage through the formation of DNA adducts. These adducts interfere with DNA replication and repair, resulting in mutations and DNA damage that drive tumor initiation and progression [28]. The 4NQO model closely resembles human OSCC in both histological and molecular characteristics, particularly in the palate and tongue, making it one of the most clinically relevant models available [30].

Moreover, the 4NQO model exhibits all stages of carcinogenesis, including initiation, promotion, and progression, and the diverse and heterogeneous nature of 4NQO-induced lesions mirrors the complexity of human OSCC [19]. Tumors can be induced in both rats and mice, either by adding low concentrations of 4NQO to their drinking water or through topical application on the tongue [31].

While new animal models for OSCC are continuously being developed, it is crucial to fully understand the characteristics, strengths, and limitations of each model. This knowledge is essential when selecting the most relevant model for preclinical studies, particularly for drug screening and therapeutic evaluation. Among the available models, the 4NQO rodent model is currently the most widely used and most representative of human OSCC [28].

In this study, we aim to provide a detailed characterization of the 4NQO rat model for OSCC, using both histological analysis and the first-ever application of ex vivo magnetic resonance imaging (MRI) to visualize tumors in this model. This innovative use of MRI enhances the clinical relevance of our study, as it mirrors the imaging modality commonly used in patients, enabling non-invasive visualization of tumor growth over time.

We evaluated two modes of 4NQO administration, topical application or the addition to drinking water, over an eight-month study period. Monthly sacrifices allowed us to track tumor development over time, identify the optimal method for OSCC induction, and correlate it with stage-dependent disease progression. Additionally, this approach enabled us to determine the minimum exposure duration required to attain sufficient tumor volumes to evaluate emerging cancer therapies. By thoroughly characterizing the 4NQO rat model, we provide valuable insights into its use in molecular and therapeutic studies on OSCC, with the ultimate goal of improving preclinical models for oral cancer research.

## 2. Materials and Methods

*Animals and tumor induction*—All animal experiments were performed according to the European Directive 2010/63/EU and were approved by the Ethical Committee for Animal Experimentation of Hasselt University (ID 201424). Eight-week-old male Wistar rats (*n* = 54) were purchased from Janvier Labs (Le Geneste-Saint-Isle, France, RRID:RGD_2308816), and were allowed to acclimatize for two weeks. Two groups received 4NQO as a carcinogen, while two groups served as controls. In the first experimental group, 4NQO was administered via the drinking water (ad libitum, 0.01 mg/mL, *n* = 27). In the second group, 4NQO was dissolved in propylene glycol (5 mg/mL) and applied topically to the tongue using a small brush while the animals were anesthetized using isoflurane, with the induction at 2–3.5% in 100% oxygen and maintenance at 1.5–3.5% at a flow rate of 1.5 L/min (3 times per week; *n* = 27). For controls, the first group received topical application of propylene glycol to the tongue (*n* = 3) to account for any potential effects of the solvent, and the second control group received no treatment (negative control, *n* = 3). The experimental unit was the individual rat; each rat was randomly assigned to a treatment group and received its respective intervention independently. Potential confounding factors such as cage location, treatment order, and timing of measurements were not systematically controlled in this study. Animals were housed under standard conditions, but cage positions were not rotated, and treatments were applied in a fixed order. This may have introduced some uncontrolled variation; however, due to the exploratory nature of the study, the primary goal was to obtain biological insight into disease progression over time. To evaluate the progression of OSCC and determine the timeline of tumor formation, three rats from each experimental group were sacrificed at monthly intervals, beginning at two months of treatment. Control animals were sacrificed at the eight-month study end point. Following sacrifice using a lethal dose of sodium pentobarbital (intraperitoneal injection; 150 mg/kg) and transcardial perfusion with 4% paraformaldehyde, ex vivo MRI scans of the heads were performed, the tongues were dissected, and tissues were processed for histological analysis by paraffin embedding.

*Histopathological examination of the tongue tissue*—For histopathological examination, the tissue was subdivided into three anatomical regions, the apex, body, and root, to evaluate the tumor development at the different anatomical locations of the tongue (Figure S1). Each region was further divided into 1000 µm subsections, from which multiple slices were prepared. Paraffin-embedded tissue slices were processed and stained using hematoxylin & eosin (H&E). H&E staining was performed automatically using the HistoCore SPECTRA Workstation (Leica, Diegem, Belgium) to evaluate pathological changes. Pathological analysis with clinic-based parameters (Figure S2) was performed by two independent surgical pathologists and an experienced researcher, all blinded to the experimental conditions. The pathologists reviewed one slice within each 1000 µm subsection and pathologically scored the subsection exhibiting the highest abnormality grade. In cases where there was disagreement in the scoring of tissue sections, an additional consultation involving all three evaluators was held to reach a consensus on the diagnosis. This qualitative assessment method aligns with current clinical practice and research standards, where independent pathological evaluation provides robust insight into key diagnostic features [32,33,34].

*Masson’s Trichome staining*—Masson’s Trichome staining was used to provide a detailed histological overview to distinguish different tissues. In short, nuclei are first visualized with Meyer’s Hematoxylin for seven minutes. After rising, Ponceau-Fuchsin was applied to highlight muscle fibers and cytoplasm. Collagen visualization was achieved using phosphomolybdic acid, followed by staining with aniline blue. Final differentiation was performed with 1% acetic acid, then sections were dehydrated, cleared in xylene, and mounted.

*Immunohistochemistry*—An immunohistochemical staining for Ki-67 was conducted to assess cellular proliferation in the tongue tissues. After rehydration, heat-mediated antigen retrieval was conducted, and a protein blocking agent (Dako, Agilent Technologies, X090, Dako, Belgium) was used for 20 min. Sections were incubated overnight at 4 °C with a primary antibody against Ki-67 (1:500, Abcam, ab15580, Cambridge, UK, RRID:AB_443209). The next day, Alexa Fluor 555-labeled goat anti-rabbit secondary antibody (1:400, Thermo Fisher Scientific, #A21430, Merelbeke, Belgium) was used together with Hoechst 33342 to visualize nuclei for 1 h (1:100, Immunochemistry technologies, #639, CA, Davis, US). Slides were mounted with Prolong Gold antifade reagent (fisher scientific, 11539306, Brussels, Belgium) and visualized with the AxioScan.Z1 slide scanner (ZEISS, Zaventem, Belgium) with imaging and analysis performed using the MAKAIA software (Fraunhofer, München, Germany).

*MRI*—MRI scans were performed from the heads of rats that were sacrificed at the above-mentioned time points. To prevent dehydration and to avoid interference with water-containing solvents, rat heads were submerged in fluorinated liquid (PFPF, fomblin^®^, Solvay, Bollate, Italy) during the MRI measurements. All MRI data were acquired on a preclinical 9.4 T MRI system (BioSpec 94/20USR, Bruker Biospin, Ettlingen, Germany) equipped with a horizontal bore magnet (20 cm bore size) and shielded gradients (up to 600 mT m^−1^). MRI scans were performed using a quadrature birdcage coil with a 72 mm inner diameter. After acquisition of a localizer scan, three orthogonal (axial, sagittal and coronal orientation) 2D T2-weighted RARE anatomical reference scans were acquired with the following acquisition parameters: RARE factor (RF)—4, repetition time (TR)—2300 ms, echo time (TE)—20 ms, in plane resolution—0.2 mm, slice thickness—1 mm and number of averages (NA)—1. Subsequently, a three-dimensional high-resolution MRI was acquired with the following parameters: Turbo-RARE sequence, RF—8, TR—2000 ms, TE—30 ms, isotropic resolution—150 µm; NA—3. MR images were processed using the Paravision software (version 5 and version 6, Bruker Biospin, RRID:SCR_001964). Images were exported in DICOM format for further analysis and quantification. MR images were displayed in a PACS system (Agfa Healthcare Enterprise Imaging, Agfa HealthCare, Belgium) and visible tumoral lesions were manually delineated, using the integrated volumetry tool. Maximal diameters of the lesions in the coronal plane and tumoral volumes were reported. Of all MRI scans, two exams were non-interpretable due to artifacts. Five datasets showed moderate image quality but the images were regarded as interpretable. All other MRI exams were of acceptable to excellent quality.

*Statistical analysis*—All statistical analyses were performed using GraphPad Prism 9 (RRID: SCR_002798). For the MRI, data were analyzed using two-way repeated measures ANOVA with Sidak’s multiple comparisons test used to compare treatment and control groups within each time point. Residuals were assessed for normality using the Shapiro–Wilk test. Due to violations of normality, the Greenhouse–Geisser correction was applied. For Ki67 staining analysis, data were assessed for normality using the Shapiro–Wilk test and a one-way ANOVA followed by Sidak’s multiple comparisons test was conducted to evaluate differences between the pathological stages. Results are expressed as mean ± SEM, and statistical significance was defined as *p* < 0.05.

## 3. Results

### 3.1. Overall Health of the Wistar Rats

Throughout this study, six animals died before reaching the study endpoint, three in the topical application group and three in the drinking water group. Interestingly, deceased rats that received 4NQO topically died before reaching seven months of 4NQO exposure all with very small to no lesions, while those in the drinking water group died after seven months of exposure to the carcinogen with visible tumor formation in both histology and MRI.

As listed in Table S1, no abnormalities were observed in the negative control or propylene glycol groups, and all animals in these groups reached their experimental endpoints. In the drinking water experimental group, CIS lesions began to form after three months of treatment (33% of animals), with all animals expressing CIS after five months. The model progresses towards ISCC by six months (100% of animals). All animals in this group that survived to the endpoint exhibited ISCC. In contrast, rats in the topical application group only began to develop CIS at approximately eight months (33% of animals), with no cases of invasive malignancies observed by the study endpoint.

### 3.2. Histopathological Analysis of the 4NQO Rat Model

**Figure 1:**
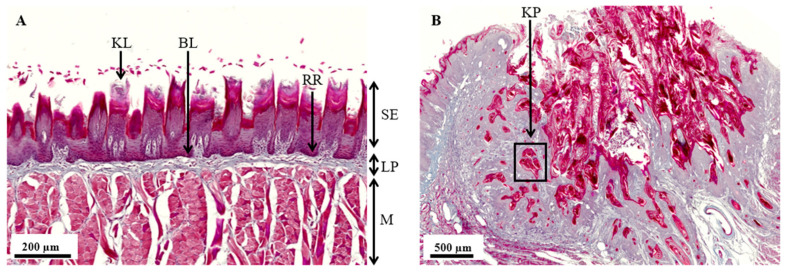
**Histological comparison of control rat tongue tissue and 4NQO-induced tumor tissue using Masson’s trichrome staining**. (**A**) Control rat tongue tissue showing its distinct histological layers. The deep muscle layer (M) comprises longitudinal, vertical, and transverse skeletal muscle fibers, with salivary glands present in the posterior third of the tongue. The lamina propria (LP) is situated above the muscle layer and contains connective tissue fibers, blood vessels, and nerves. The uppermost stratified epithelium (SE) layer includes the basal layer (BL), where OSCC typically originates, followed by multiple squamous cell layers and a keratinized layer (KL), which is characteristic of rat tongue tissue. The regular formation of rete ridges (RRs) is a notable feature of the tongue epithelium. Scale bar = 200 µm. (**B**) Rat tongue tissue after 6 months of 4NQO exposure via drinking water shows an invasive squamous cell carcinoma. The normal tissue architecture is disrupted, with the tumor invading the underlying layers. The formation of keratin pearls (KPs), indicative of squamous differentiation, is a hallmark of these well-differentiated tumors. Scale bar = 500 µm.

The normal histological structure of an untreated rat tongue is shown in Figure 1A. It is organized into three distinct layers: the upper stratified squamous epithelium (SE), the middle lamina propria (LP), and a deep muscular layer (M). The SE consists of highly organized layers of flattened epithelial cells, with the superficial portion showing pronounced keratinization, reflecting the consumption of harder, unprocessed food by the rat. This contrasts with the limited keratinization seen in humans, who typically consume softer, cooked foods. The basal lamina (BL), where OSCC typically originates, connects the oral epithelium with the lamina propria, which is a dense, flexible connective tissue layer rich in blood vessels, nerves, and lymphatic vessels, supporting sensation and nutrition. The underlying muscle layer of the rat tongue is composed of several distinct layers of muscle fibers, each oriented in a specific direction, facilitating a wide range of tongue movements.

Figure 1B shows an ISCC in a drinking water 4NQO-exposed rat tongue, where the normal tissue architecture is severely disrupted. Hallmarks of ISCC include pronounced keratinization due to the production of keratin by tumor cells, often leading to the formation of keratin pearls (KPs). These keratin pearls are a defining characteristic of squamous cell differentiation within the tumor, indicating advanced disease progression.

**Figure 2:**
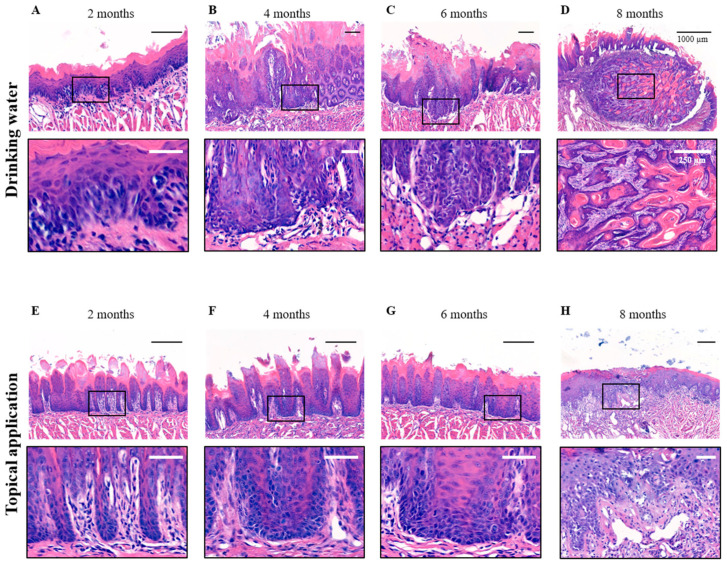
**Histological analysis of H&E-stained tongue epithelium following eight months of 4NQO exposure.** Representative histological images of the tongue epithelium following 4NQO administration via drinking water and topical application after two, four, six, and eight months of treatment. (**A**) At two months, low-grade dysplasia is observed, progressing towards (**B**) medium-grade dysplasia after 4 months. (**C**) By 6 months, carcinoma in situ has developed, and by (**D**) 8 months, the tumor has invaded the underlying tissue layers, forming ISCC and sporadic keratin pearls. Scale bar (black) = 100 µm in overviews; scale bar (white) = 50 µm in zooms unless otherwise noted in the figure (right panels). (**E**) Following topical application of 4NQO, reactive tissue was observed after two months, (**F**) developing to low/medium-grade dysplasia after 4 months. (**G**) This dysplastic stage persists throughout the remaining exposure period, with (**H**) CIS formation occurring in one-third of the cases after the eight-month endpoint. Scale bar (black) = 200 µm in overviews; scale bar (white) = 50 µm in zooms.

The development of OSCC is influenced by both the method of 4NQO administration and the specific location on the tongue (Figure S1). To assess these differences, we compared histological outcomes (listed in Figure S2) following 4NQO administration via drinking water and topical application using H&E staining (Figure 2). After two months, both administration methods preserved the distinct histological features of the rat oral epithelium (Figure 2A,E). By four months of 4NQO administration in the drinking water, moderate dysplasia was observed in all animals, characterized by disorganized cell growth within the lower and middle thirds of the epithelial layer (Figure 2B). Abnormal cells displayed nuclear enlargement and irregular nuclear contours, with a loss of normal cell polarity resulting in a disordered and crowded cell arrangement. Delayed maturation of cells towards the oral cavity was observed, and irregular, bulbous rete ridges contributed further to the disordered architecture. After six months of 4NQO in the drinking water, the epithelial structure was completely disrupted, leading to the formation of CIS (Figure 2C). Dysplastic changes extended throughout the entire layer of the epithelium, with abnormal cells spanning from the basal layer to the epithelial surface. After eight months, the dysplastic epithelium invaded the underlying tissue layers in all animals, progressing to an ISCC characterized by the formation of keratin pearls, a hallmark of squamous differentiation (Figure 2D).

In contrast, with topical 4NQO application to the tongue, after two months, only reactive tissue was observed (Figure 2E), which refers to non-cancerous changes in response to stress or injury, progressing towards low- to medium-grade dysplastic tissue after four to six months (Figure 2F,G). However, after eight months, CIS was found in some of the animals (33%) (Figure 2H), though no invasive malignancies were noted at the study endpoint.

To precisely determine where tumors develop on the tongue following 4NQO exposure, the tissue was subdivided into three anatomical regions: the apex, body, and root. This approach allowed detailed analysis of tumor progression in each specific region. Following 4NQO administration through drinking water, distinct patterns of tumor development were observed across these regions (Table 1A).

After two months, rat tongues exhibited reactive epithelium with low to moderate squamous dysplasia (SD) in some cases. Notably, flat dysplasia was predominantly observed in the apex and root, while the body exhibited a more papillary growth pattern. By the third month, CIS had developed in one out of three rats, progressing to CIS in 100% of the animals by five months. ISCC was first detected in the tongue body at six months, with the apex and root developing ISCC at seven months in all animals. ISCC was generally well-differentiated and predominantly expanding inwards, invading underlying tissue layers, called endophytic tumor growth. The pattern of invasion varied, ranging from a pushing border to a more cord-like invasive pattern.

In contrast, when 4NQO was applied topically to the tongue, tumor development occurred more slowly and was less frequent (Table 1B). The tissue remained largely reactive for the first two months, with low-grade dysplasia emerging in the third month. From four to seven months, the rats developed low- to medium-grade SD, with CIS observed in one of three animals by the eighth month. The grade of dysplasia exhibited considerable heterogeneity throughout the treatment period.

These contrasting outcomes between administration routes led us to examine whether differences in effective carcinogen exposure could explain the observed dissimilarity in tumor onset and severity. Although the topical group received a higher nominal concentration of 4NQO (5 mg/mL), the small application volume (~25 µL), reduced frequency (three times per week), and partial loss due to behaviors like spitting resulted in a lower cumulative dose of approximately 1.63 mg of 4NQO per month. In contrast, the drinking water group received a lower concentration (0.01 mg/mL) but had continuous access, resulting in a cumulative monthly 4NQO intake of approximately 7.61 mg. These findings emphasize that not only concentration, but also the route, frequency, and duration of exposure have a significant influence on tumorigenesis. Importantly, these two administration routes mimic different human exposure scenarios: localized, acute exposure (e.g., chewing tobacco) versus chronic, systemic exposure (e.g., cigarette smoking).

Additionally, parakeratosis was noted in both models, regardless of the stage or location. Although lymphocytic inflammation was observed in both 4NQO exposure groups across all stages, its presence is rather limited.

**Figure 3:**
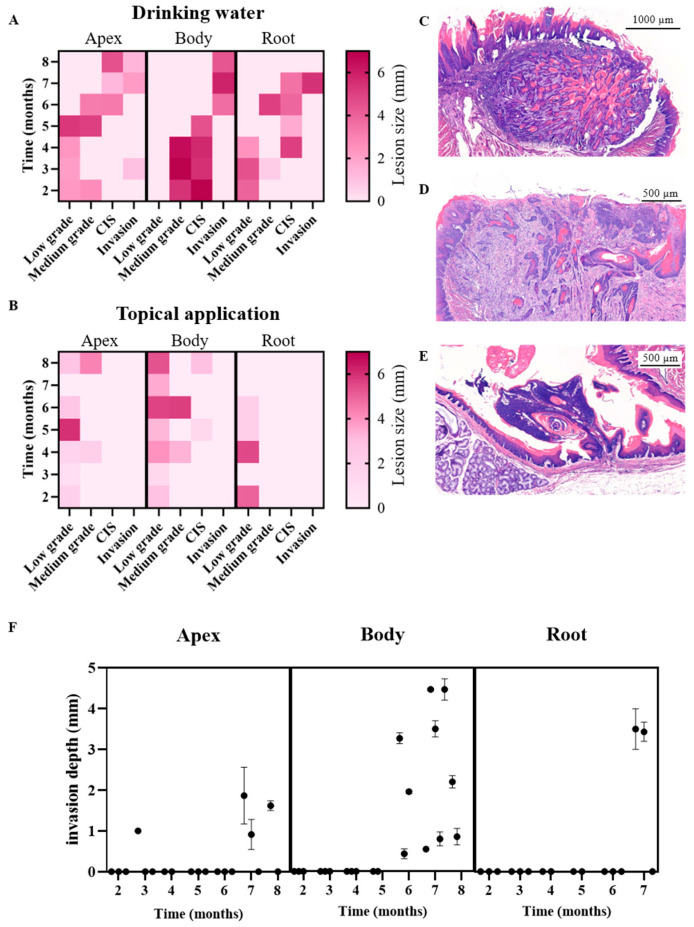
4NQO-induced lesion size and invasion patterns differ in both application methods over time for the different tongue regions. Heatmap representing the lesion size (in mm) induced by 4NQO in different regions of the tongue (apex, body, root) in rats that received (**A**) drinking water or (**B**) topical application over a time period of eight months. Moreover, the invasion pattern is heterogeneous. (**C**) Endophytic tumor with a pushing border, where the underlying tissue layers are compressed rather than extensively infiltrated. Scale bar = 1000 µm. (**D**) Infiltrative growth pattern, where the tumor invades the lamina propria and muscle layer in strands and cords of cells, indicative of advanced, aggressive cancer. Scale bar = 500 µm. (**E**) Exophytic growth pattern, characterized by outward proliferation of tumor cells, resulting in a mass projecting above the tissue surface. This growth contrasts with infiltrative patterns as the tumor primarily expands outward before deeper tissue invasion. Scale bar = 500 µm. (**F**) Time-dependent variation in invasion depth based on tongue region for 4NQO administration via drinking water. With topical 4NQO administration, no invasive growth was detected. Data are represented as mean ± SEM, where SEM reflects the subjective assessments of three independent evaluators.

The lesion size of 4NQO-administered rats was also compared for both application methods with the lesion diameter determined over time for each region of the tongue. In the drinking water group (Figure 3A), lesions at the apex of the tongue exhibited an increase in size, reaching medium-grade dysplasia by five months. However, upon progression to CIS at six months, lesion size decreased, with a further reduction observed at the formation of ISCC. In the body of the tongue, the largest lesions were noted and classified as medium-grade dysplasia or CIS from two months onward. Following the development of the invasive tumor at six months, the lesions initially appeared smaller but subsequently enlarged over time. At the root of the tongue, lesion size displayed rather heterogeneous characteristics over the eight-month observation period and across various stages of dysplasia.

In rats that received 4NQO topically (Figure 3B), we generally observed smaller lesions compared to the drinking water group. In the apex of the tongue, lesion size increased over time but remained classified as low- to medium-grade dysplasia for up to eight months. Although lesions in the body of the tongue were larger, they did not progress to ISSC. However, CIS was observed, albeit with small lesion sizes. At the root of the tongue, lesions following topical application were small and classified as low-grade dysplasia. Overall, lesions induced by 4NQO in drinking water were larger, more homogeneous, and progressed to ISCC in contrast to those induced by topical application.

The invasive growth pattern of OSCC in the 4NQO rat model demonstrated considerable variability and could be classified into distinct subtypes. Tumors with an endophytic growth pattern could be further subdivided into two subtypes: tumors with a pushing border (Figure 3C) and tumors exhibiting infiltration in cords and strands (Figure 3D). Tumors with pushing border displayed a smooth and distinct boundary, compressing and displacing surrounding tissues without irregular infiltration. In contrast, infiltrating tumors grew irregularly, invading surrounding tissues as narrow strands, cords, or small clusters of neoplastic cells.

Additionally, exophytic tumor masses were also observed (Figure 3E). These tumors expanded outwards, away from the underlying tissue, making them more readily visible on the surface and easier to detect macroscopically. Unlike endophytic tumors, exophytic growth did not invade deeper layers but remained superficial.

The depth of tumor invasion varied based on both the duration of 4NQO treatment and the anatomical region of the tongue (Figure 3F). In the apex, invasive growth was detected from seven months onwards in animals exposed to 4NQO in drinking water. In the body of the tongue, invasion was detected after five to six months in the drinking water group, with increasing invasion depth observed over time. The root of the tongue, however, showed invasive growth starting from six and a half months onwards with administration of 4NQO in drinking water. Interestingly, no invasion was observed in the tongue of animals with topical 4NQO administration, indicating that this method of administration resulted in a delayed and less invasive progression compared to drinking water exposure.

### 3.3. High Cell Proliferation in Dysplastic Lesions

**Figure 4:**
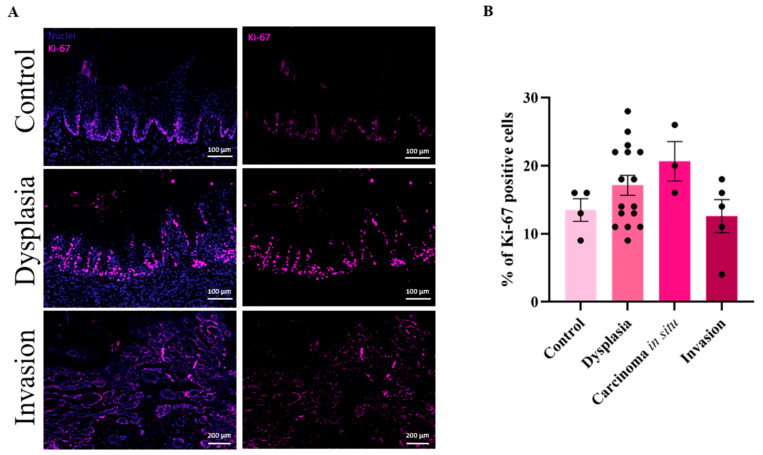
**Increased cell proliferation in dysplastic stages of 4NQO-induced OSCC.** (**A**) Ki-67, a marker of cell proliferation, was used to assess proliferation in control rat tongues and in 4NQO-induced dysplastic and invasive lesions. Scale bar control and dysplasia = 100 µm; scale bar invasion = 200 µm. (**B**) Quantification of the percentage of Ki-67 positive cells across different stages of OSCC progression. A marked, yet non-significant increase in Ki-67-positive cells was observed in dysplastic lesions compared to controls, while a decline in proliferation was noted upon the development of invasive tumors. Data are represented as mean ± SEM (*N* ≥ 3).

The proliferative dynamics of 4NQO-induced malignancies were analyzed to gain deeper insights into tumor progression from dysplasia to invasive OSCC. As shown in Figure 4A, Ki-67, a marker of cellular proliferation, exhibited a marked, yet non-significant increase in dysplastic tissue compared to control rat tongues, highlighting the elevated proliferative activity during early tumorigenesis. Interestingly, this increased proliferation diminished in the invasive OSCC stage, suggesting a shift in tumor biology. Quantification of Ki-67-positive cells (Figure 4B) further confirmed these findings. Control tissues presented less than 15% of Ki-67 positive cells, whereas proliferation progressively increased through the stages of dysplasia and CIS. Notably, proliferation declined upon the transition to invasive OSCC. These results suggest that early dysplastic lesions are characterized by uncontrolled cell proliferation, which likely drives tumor formation [35]. However, as the tumor evolved into an invasive state, a reduction in proliferative activity occurred, possibly indicating a shift from rapid cell division towards enhanced invasive capacities. This shift underscores the complex interplay between proliferative and invasive behaviors during malignant progression.

### 3.4. MRI Successfully Detects Oral Malignancies After 4NQO Treatment in Rats

**Figure 5:**
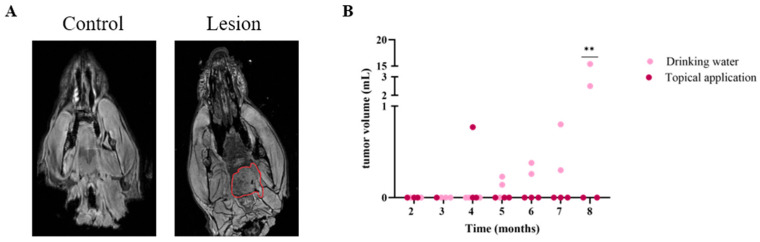
**OSCC is visualized with ex vivo MRI.** (**A**) Representative ex vivo MRI images displaying the head of a control rat (left) and lesion formation on the tongue of a rat after 4NQO treatment (lesion demarcated in red). (**B**) Comparison of lesion development between two 4NQO application methods. In the drinking water group, lesions were consistently observed starting from 5 months of treatment. In contrast, topical application failed to induce tumor formation, except for a single small lesion detected at 4 months. At 8 months, a significantly increased tumor volume was observed in the drinking water group compared to topical application. Data are represented as individual data points. ** *p* ≤ 0.01.

To assess the feasibility of non-invasive imaging and its alignment with histological analysis, ex vivo MRI was conducted in the 4NQO rat model. This method provided crucial insights into malignancy development and offered a distinct advantage by enabling the evaluation of tumor progression across the entire tongue, including lesion count and volume, unlike histology which is limited to selected regions. Additionally, when MRI analysis is performed prior to tissue sectioning, it can guide targeted and in-depth histological evaluations, further enhancing the precision of pathological analysis in this model. Figure 5A shows representative MR images from a control animal (left) and an annotated lesion (right) after 4NQO application via drinking water. The animals of the 4NQO model with drinking water administration showed significantly larger tumor volumes on MRI, compared to the animals with topical 4NQO applications (Figure 5B). Except for one animal in the 4NQO drinking water group, all scans of the animals in the 4NQO drinking water group showed a macroscopic tumoral lesion on the tongue from 5 months onwards. However, tumor volumes were variable, between 2.5 mL and 15.4 mL at eight months of treatment. In the animal group with topical application, no macroscopic tumoral lesions were visible on MRI. Interestingly, all lesions were visible in the posterior part of the tongue. Three MRI scans demonstrated bifocal lesions and four lesions showed hemorrhagic foci. In addition, no large tumors were observed in other parts of the rat mouths.

## 4. Discussion

The 4NQO carcinogen has been widely used over the years to induce OSCC in both mice and rats. Rats specifically are a valuable model to study oral cancers due to their anatomical and physiological similarities to humans, which enable researchers to draw meaningful parallels in the development and progression of these cancers. Additionally, rats are easy to maintain and breed, and their short lifespan facilitates the study of long-term effects of treatments and interventions within a relatively brief period. Their well-established immune system is crucial to examine cancer progression and responses to therapies, enabling the investigation of immune interactions with cancer cells and potential immune-targeted treatments. Moreover, the extensive use of rats in oral cancer research provides a robust foundation of existing knowledge and experimental tools that researchers can leverage in their studies [17,36].

Despite the common use of the 4NQO model in Wistar rats for OSCC research, Zigmundo et al. highlighted several experimental limitations, stressing the need for a more detailed characterization [37]. To the best of our knowledge, no previous study has compared the topical application of 4NQO with its administration via drinking water in rats. Similar comparisons have been conducted in mice, with findings that are in line with our results. For instance, the study of Tang et al. reported a higher incidence of oral carcinogenesis when 4NQO was administered through drinking water compared to topical application [38]. Nevertheless, the majority of the available rat studies rely on endpoint measurements, missing crucial stages in carcinoma development [37]. Our study provides a comprehensive time- and application-dependent analysis of OSCC formation in 4NQO-treated rats, enhancing this model for future research.

Numerous studies describe 4NQO topical administration for the induction of OSCC, with heterogeneous results [38]. In our research, epithelial dysplasia varies from low- to medium-grade, with a flat or papillary structure independent of location and timing. Moreover, CIS is observed after eight months, whereas in most studies, malignancies are noted after five to seven months. These studies, however, employed a higher dose (0.5%) of 4NQO and applied it to the oral palate rather than the tongue of the animals [39,40,41]. Nevertheless, the tongue represents a more relevant target, exhibiting the highest incidence of OSCC [42]. In order to correctly compare both application methods, we also chose to topically apply the carcinogen on the tongue rather than the oral palate, as delivering the carcinogen via drinking water was reported to induce cancer in the dorsum of the tongue [43]. The topical application of 4NQO both on palate and tongue has several disadvantages. The process is labor-intensive because it requires frequent handling of the animals. Moreover, rats need to be anesthetized multiple times a week, which increases stress and risk to the animals. Notably, at certain points, animals start spitting out the 4NQO substance, explaining the low incidence of CIS and the tumor formation on the apex of the tongue [38]. Additionally, systemic toxic effects after 4NQO topical application in the liver, spleen, and kidneys are reported by Barcessat et al. [44].

Administration of 4NQO in drinking water, on the other hand, became the most conducted method in the last few years. Compared to topical application, administering 4NQO through the drinking water of rodents offers several advantages. As seen in our results, tumor induction is more consistent with early dysplastic phases, featuring a flat SD at the root and apex of the tongue and a more papillary development in the body of the tongue. In later phases, CIS is formed starting in the middle part of the tongue, probably related to the way rats drink water [43]. Pathological analyses of 4NQO-induced mouse models via drinking water similarly show a flat SD, exophytic papillomas, and invasive squamous cell carcinoma [38]. Moreover, the model mimics the carcinogenesis process due to an even exposure of the entire oral mucosa to the carcinogen, reflecting the clinical situation. Additionally, the systemic method requires lower concentrations of 4NQO, reducing the risk of overexposure while still ensuring effective oral cancer development [37,38,45]. Despite the toxicity of 4NQO in different organs, the administration of lower concentrations in drinking water results in tongue-specific squamous cell carcinoma [46]. Compared to previous studies, CIS was observed after three months of 4NQO exposure whereas the group of Dayan only reported CIS formation after five months with an identical dose of 4NQO [47,48,49]. However, in our study, although CIS was seen starting from three months, it also took five months for 100% of the animals to display CIS. Although several studies use higher 4NQO doses [43,50,51,52], it is important to take the cytotoxicity into account.

Our study highlights that not just the concentration of 4NQO, but the route, frequency, and cumulative dose of exposure critically influence tumor development. Despite receiving a lower nominal concentration, animals in the drinking water group developed earlier, larger, and more invasive lesions compared to the topically exposed group. These findings suggest that chronic, systemic exposure, as modeled by the drinking water protocol, more effectively replicates the multistep nature of human OSCC progression, and thus provides a more translationally relevant platform for preclinical research.

Moreover, systemic exposure in models like the 4NQO drinking water protocol allows researchers to investigate systemic and immune-related effects, which are increasingly recognized as key contributors to oral carcinogenesis [53,54]. For example, chronic exposure via drinking water has been associated with immune cell death and systemic inflammatory alterations, phenomena that are difficult to fully capture in purely localized topical models. Notably, inflammation in the tongue was generally limited in our histological analyses for both topical application and drinking water. In previous research investigating the effects of 4NQO on immune cells, a decrease in certain lymphocyte populations was detected in the spleen and in the peripheral blood circulation, indicating the suppression of the immune system during 4NQO-induced tumor development. Growing evidence highlights the importance of the immune microenvironment on tumor progression. Specifically, recent studies indicate that 4NQO exposure can induce early immunosuppression by promoting immune cell death, thereby contributing to a chronic inflammatory microenvironment that supports tumor progression [53]. Furthermore, OSCC is widely recognized as an immunosuppressive disease. Tumor cells employ multiple mechanisms to evade immune surveillance, including the accumulation of immunosuppressive cytokines (e.g., IL-10, TGF-β), impaired cytotoxic function, reduced antigen presentation, and T-cell exhaustion [55]. In addition, previous work has shown that cytokines and cytokine receptors are involved in the development of oral malignancy, alongside early induction of Cox2 and NF-κB activation during the initial stages of 4NQO treatment [56]. These pathways are known to contribute to inflammation-driven tumor progression and highlight the importance of inflammatory and metabolic alterations in tumor tissues during oral carcinogenesis. While detailed immune and cytokine profiling was beyond the scope of this study, this presents an important aspect for future studies.

OSCC exhibited diverse invasive growth patterns, classified as either endophytic, with pushing borders or infiltrative patterns, or exophytic, characterized by superficial, outward growth. Notably, the drinking water administration method resulted in a higher prevalence of endophytic tumors [43], providing a useful model to study more aggressive and invasive tumor behavior. This is particularly relevant given the clinical significance of endophytic tumors, which are associated with worse patient outcomes and increased pain due to their deeper tissue invasion [57,58,59]. The ability to replicate such tumor types in the 4NQO model offers valuable insights into the mechanisms driving invasion and progression, and highlights the importance of focusing research on these more aggressive growth patterns to improve prognosis and therapeutic strategies. The depth of invasion depended on the method and duration of 4NQO exposure. Drinking water administration led to earlier and deeper invasion, particularly in the body and root of the tongue, while topical application resulted in delayed, less invasive progression. These findings suggest that the method of 4NQO exposure significantly influences the aggressiveness and invasion depth of OSCC. In our study, drinking water leads to more invasive tumors compared to topical application under the tested concentrations and frequency. Moreover, the results showed considerable heterogeneity in both lesion size and invasion depth among animals sacrificed at the same time point, a variation that was also clearly captured by ex vivo MRI. These findings emphasize not only the relevance of longitudinal non-invasive imaging to accurately monitor tumor progression in this model, but also its broader applicability in preclinical cancer research. The capacity to detect such differences early and across multiple animals enhances the clinical relevance of the 4NQO model, as it closely reflects the heterogeneity observed in human OSCC patients [60,61]. Interestingly, invasive OSCC was identified histologically after five months of treatment, coinciding with the time point at which MRI was able to detect lesions. This imaging technique, which closely mimics clinical methods [62], provided critical insights into the development and progression of malignancies, enhancing the translational relevance of the model. In our study, we chose to use MRI ex vivo due to the duration of the high-resolution scans (>2 h) and to avoid movement artifacts. In future in vivo MRI studies with clinically used contrast agents, longitudinal follow-up of tumor development in individual animals would be possible. Moreover, MRI provides crucial guidance for histological analysis, streamlining pathological evaluation by directing attention to specific tumor regions, thereby accelerating the assessment process.

Ki-67 expression patterns provided important insights into cell proliferation during the progression of oral carcinogenesis. We observed elevated Ki-67 expression starting in low-grade dysplasia, which is consistent with early cellular proliferation as tissue begins to undergo malignant transformation. High Ki-67 expression persisted in medium-grade dysplasia and CIS, reflecting the continued rapid cell division in these pre-invasive stages of tumor development. Interestingly, Ki-67 expression decreased in invasive carcinomas. This reduction suggests a shift in the tumor biology once invasion occurs. While early-stage lesions, such as dysplasia and CIS, are marked by high proliferative activity, invasive OSCC may exhibit more heterogenous cell cycle dynamics. Reduced Ki-67 expression in invasive tumors could indicate that, although invasive cancers remain aggressive, their proliferative index might be reduced compared to earlier stages, possibly due to altered growth patterns, tumor microenvironmental factors, or the emergence of more quiescent or resistant cancer cell populations [63]. These findings align with previous reports showing that Ki-67 is a robust marker for early tumor proliferation but may not fully reflect the complexity of invasive cancer behavior [64,65]. The lower proliferation rates in invasive tumors might also suggest a transition to a more invasive, yet less rapidly dividing phenotype, possibly driven by other mechanisms such as increased migratory or metastatic potential rather than pure proliferation [63]. While Ki67 stainings are a valuable tool to assess cell proliferation, fluorescence stainings may not fully capture the complexity of tumor biology, as it does not allow the distinction between proliferating tumor cells and non-tumor cells [66].

The 4NQO drinking water model for OSCC offers several advantages, including its ability to closely mimic the clinical presentation of oral cancer, with diverse and heterogeneous lesions that reflect human disease. However, one key limitation is its lacking immune response, which makes it less ideal to study the immunological aspects of cancer biology. This is a significant drawback given that OSCC are among the most immune-infiltrated cancer types. Research has demonstrated that immune patterns, responses, and resistance mechanisms are critical in the development and progression of OSCC, making the tumor immune microenvironment a promising therapeutic target [67]. Additionally, it does not fully capture the genetic or epigenetic predispositions that contribute to OSCC in humans. While it reflects the environmental carcinogenesis aspect, human OSCC often involves a combination of environmental exposures and genetic mutations or alterations. This limitation means that the model may not accurately represent all cases of OSCC, particularly those influenced by inherited risk factors or genetic syndromes. A potential improvement would be the integration of the 4NQO model with transgenic mouse models. Combining chemical carcinogenesis with genetically engineered mice that carry mutations relevant to OSCC could better reflect the multifactorial nature of the disease, including the interaction between environmental and genetic factors. This hybrid approach would more closely mimic the pathogenesis of human OSCC, especially in individuals with genetic susceptibilities combined with carcinogen exposure, such as those with chronic tobacco and alcohol use [19].

## 5. Conclusions

Our study highlights the effectiveness of the 4NQO rat model in mimicking the full tumor progression of OSCC, particularly when administered via drinking water. This method leads to an earlier onset of CIS, larger lesions, and deeper invasion, closely resembling human OSCC. The detailed regional analysis of the tongue, the histopathological findings and the MRI images emphasize that OSCC predominantly forms in the body, more towards the root of the rat tongue. This in-depth characterization of the 4NQO model shows its value for translational research, providing a robust platform for preclinical testing of novel OSCC therapeutics.

## Figures and Tables

**Table 1:** **Comparison of OSCC formation over time in the topical application method and via the drinking water method.** (A) When 4NQO is added to the drinking water of rats, the animals show a dysplastic epithelium starting from two months onwards. After three months, carcinoma in situ is already observed, progressing towards an invasive carcinoma from six months onwards. (B) Topical application of 4NQO shows low-grade dysplasia throughout the whole model, only evolving to moderate dysplasia after four months. CIS is observed after eight months; however, no invasive carcinoma was detected. SD: squamous dysplasia. *n* = 3 for each condition at each time point.

A	2 Months	3 Months	4 Months	5 Months	6 Months	7 Months	8 Months
Drinking water	Reactive						
	Low-grade dysplasia						
	Medium-grade dysplasia						
		Carcinoma in situ					
					Invasive carcinoma		
Apex	Flat SD	Flat SD	Flat and papillary SD	Flat SD	Flat and papillary SD	Well- differentiated, exophytic and endophytic (pushing border) invasion	Well- differentiated, exophytic and endophytic (pushing border) invasion
Body	Papillary SD	Papillary SD	Papillary SD	Papillary SD	Well-differentiated, endophytic (pushing border and infiltrative) invasion	Well-differentiated, endophytic (pushing border and infiltrative) invasion	Well-differentiated, endophytic (pushing border and infiltrative) invasion
Root	Flat SD	Flat and papillary SD	Flat SD	Flat SD	Papillary and endophytic SD	Well-differentiated, endophytic (infiltrative) invasion	
**B**	**2 Months**	**3 Months**	**4 Months**	**5 Months**	**6 Months**	**7 Months**	**8 Months**
Topical application	Reactive						
	Low-grade dysplasia						
			Medium-grade dysplasia				
							Carcinoma in situ
Apex	No hyperplasia	Flat SD	Flat SD	Papillary SD	Papillary SD		Flat SD
Body	Flat SD	Flat SD	Papillary SD	Flat SD	Papillary SD	Flat SD	Papillary and endophytic SD
Root	Flat SD	Flat hyperplasia	Papillary SD	Flat SD	Flat SD		Flat SD

## Data Availability

The data generated in this study are available upon request from the corresponding author.

## Supplementary Materials

The following supporting information can be downloaded at https://www.mdpi.com/article/10.3390/cancers17132108/s1: Figure S1: Subdivisions of rat tongue for histopathological examination; Figure S2: Overview of the used parameters in the histo-pathological examination of the 4NQO rat tongues.; Table S1: Comparison of 4NQO-induced OSCC development via topical application and addition to the drinking water.

## Abbreviations

The following abbreviations are used in this manuscript:

OSCC	Oral squamous cell carcinoma
CIS	Carcinoma in situ
ISCC	Invasive squamous cell carcinoma
DMBA	9,10 dimethyl-1,2,benzanthracene
4NQO	4-Nitroquinoline 1-oxide
MRI	Magnetic resonance imaging
H&E	Hematoxillin & eosin
SE	Squamous epithelium
LP	Lamina propria
M	Muscular layer
BL	Basal lamina
KPs	Keratin pearls
SD	Squamous dysplasia
