# Supplementary material for: Optimizing Preclinical Models for Oral Cancer: The Influence of 4NQO Administration Routes on Tumor Development
Source: Cancers (Basel). 2025 Jun 23;17(13):2108. doi: 10.3390/cancers17132108 (PMC12249073; doi:10.3390/cancers17132108)
Supplement: Supplementary file 1 [file cancers-17-02108-s001.zip › cancers-3670123-supplementary.pdf]

Supplementary Material: Optimizing preclinical models for oral cancer: influence of 4NQO administration routes on tumor development

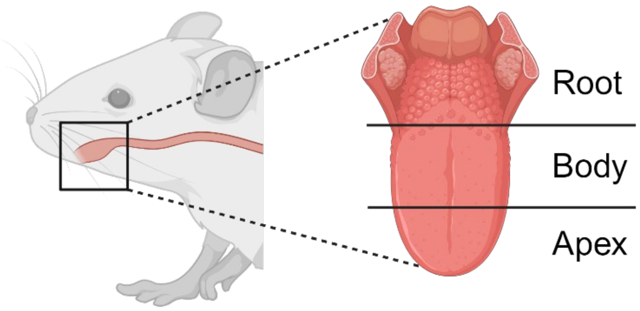

Figure S1: Subdivisions of rat tongue for histopathological examination. *To map the location of tumor development and location, the tongue of each rat is divided into different regions. The top of the tongue is called the apex, the middle is described as the body and the posterior part is the root of the tongue, connected to the pharynx. Created in BioRender. https://BioRender.com/r83p696*

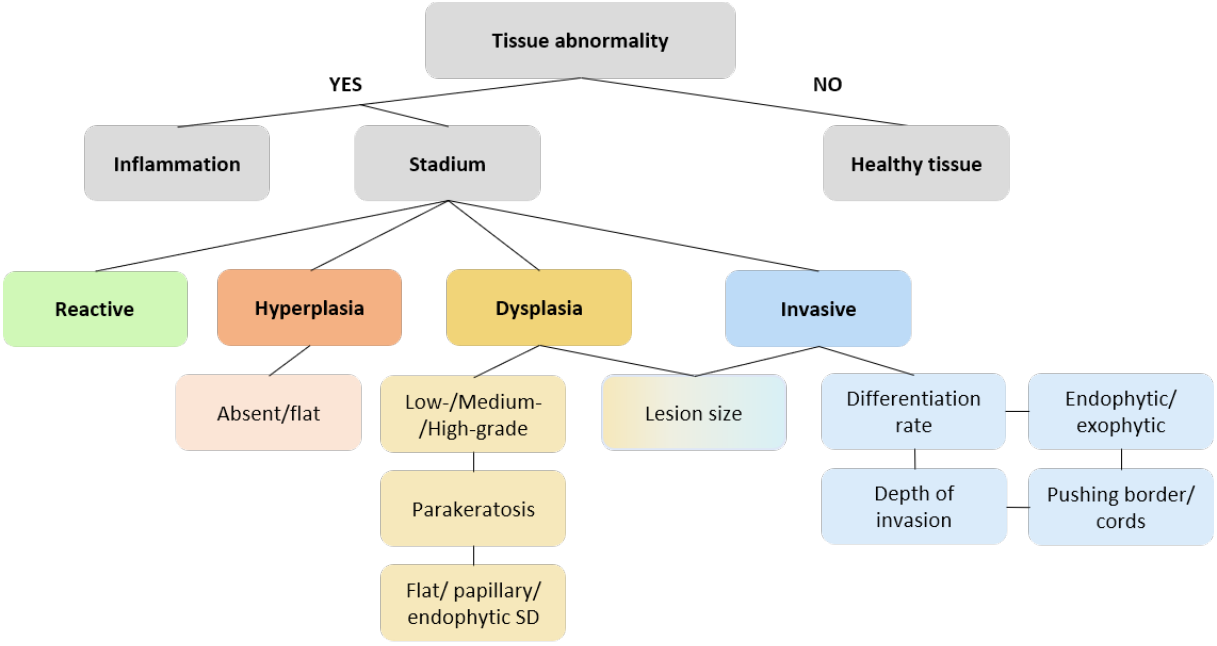

Figure S2: Overview of the used parameters in the histopathological examination of the 4NQO rat tongues. *Surgical pathologists and an experienced researcher scored all rat tongues based on different clinically relevant parameters. Inflammation state and stadium of the tumor formation were assessed in detail to provide a complete overview of the OSCC development in the 4NQO model with both application methods through time. SD: squamous dysplasia*

Table S1: Comparison of 4NQO-induced OSCC development via topical application and addition to the drinking water. *Rats were exposed to 4NQO for a total of eight months. Starting from two months of exposure, three animals per group were sacrificed monthly to monitor tumor development and progression. Tongues were dissected, embedded, stained with H&E, and analyzed by two independent pathologists and an expert researcher. The table compares tumor development over time between the two administration methods. For each time point, N=3 per group. CIS: carcinoma in situ; ISCC: invasive squamous cell carcinoma.*

**Method**	**4NQO concentration**	**Treatment time**	**Frequency of treatment**	**No. of rats**	**No. of deaths**	**Start of CIS formation**	**Start of invasion**	**Incidence of CIS at endpoint**	**Incidence of invasive SCC at endpoint**
Negative control				3	0	NA	NA	0%	0%
Propylene glycol control				3	0	NA	NA	0%	0%
Drinking water	0.1 mg/mL	8 months	*Ad libitum* in drinking water	24	3	3 months	6 months	0%	100%
Topical	5 mg/mL	8 months	3 times/week	24	3	8 months	NA	33%	0%
